# Co-prescription network reveals social dynamics of opioid doctor shopping

**DOI:** 10.1371/journal.pone.0223849

**Published:** 2019-10-25

**Authors:** Brea L. Perry, Kai Cheng Yang, Patrick Kaminski, Meltem Odabas, Jaehyuk Park, Michelle Martel, Carrie B. Oser, Patricia R. Freeman, Yong-Yeol Ahn, Jeffery Talbert

**Affiliations:** 1 Network Science Institute, Indiana University, 1001 45/46 Bypass, Bloomington, IN, United States of America; 2 Department of Sociology, Indiana University, Bloomington, IN, United States of America; 3 School of Informatics, Computing, and Engineering, Indiana University, Bloomington, IN, United States of America; 4 Department of Psychology, University of Kentucky, Lexington, KY, United States of America; 5 Department of Sociology, University of Kentucky, Lexington, KY, United States of America; 6 Department of Pharmacy Practice and Science, University of Kentucky, Lexington, KY, United States of America; University of Miami, UNITED STATES

## Abstract

This paper examines network prominence in a co-prescription network as an indicator of opioid doctor shopping (i.e., fraudulent solicitation of opioids from multiple prescribers). Using longitudinal data from a large commercially insured population, we construct a network where a tie between patients is weighted by the number of shared opioid prescribers. Given prior research suggesting that doctor shopping may be a social process, we hypothesize that active doctor shoppers will occupy central structural positions in this network. We show that network prominence, operationalized using PageRank, is associated with more opioid prescriptions, higher predicted risk for dangerous morphine dosage, opioid overdose, and opioid use disorder, controlling for number of prescribers and other variables. Moreover, as a patient’s prominence increases over time, so does their risk for these outcomes, compared to their own average level of risk. Results highlight the importance of co-prescription networks in characterizing high-risk social dynamics.

## Introduction

Prescription drug abuse is an unmitigated public health crisis that has been developing for decades [1], despite increasing regulatory efforts. Drug overdose was responsible for more than 70,000 deaths in the U.S. in 2017, making the current drug epidemic the deadliest in history [2]. The Centers for Disease Control (CDC) reports that opioids are the leading cause of overdose mortality, and more than 40% of opioid overdoses are attributable to prescription opioids [3].

One of the primary means of obtaining prescription opioids is through “doctor shopping” [4], or soliciting prescriptions for controlled substances from multiple clinicians by over-reporting or manufacturing symptoms. Among those with prescription drug dependence, nearly 40% are estimated to engage in doctor shopping [5]. Moreover, compared to people who abuse prescription drugs but do not shop doctors, doctor shoppers are more likely to experience drug-related hospital admission [6], non-fatal opioid overdose [7], and opioid overdose fatalities [8,9]. Thus, doctor shopping is a critical mechanism that contributes to opioid misuse trajectories [7].

In response to the increasing prevalence of prescription drug abuse and diversion, all fifty states and Washington D.C. have created prescription drug monitoring programs (PDMPs) to discourage doctor shopping and to reduce inappropriate prescribing and dispensing [10]. While PDMP implementation has recently been associated with modest decreases in rates of prescription opioid prescribing, diversion, and poisoning in some geographic areas, evidence overall is mixed [11]. Moreover, the U.S. continues to see increases in opioid dependence and related morbidity and mortality [12–15]. These findings suggest that although PDMPs may have reduced egregious drug-seeking and prescribing behavior, they have not addressed opioid misuse initiation and early patterns of abuse that fall short of detection criteria used to identify fraudulent behavior.

Doctor shopping has historically been difficult to characterize [15]. A common method of measurement uses multiple provider episodes (MPE), defined as obtaining controlled substances from some minimum number of prescribers and/or pharmacies in a given period of time. Although commonly used MPE thresholds have low false positive rates [7, 16, 17], this approach is crude and may be overly conservative. For example, people who engage in low levels of doctor shopping or those who doctor shop heavily for a brief period are unlikely to be correctly classified. Therefore, it is critical to explore alternative methods for characterizing prescription drug seeking behaviors to facilitate early intervention and prevention.

Recent research suggests that social processes may be a critical element of doctor shopping [16, 18, 19]. First, doctor shopping is clustered around particular at-risk prescribers. Doctor shoppers systematically seek out physicians who are complicit, easily manipulated, or unlikely to monitor electronic data [19]. For instance, a previous study estimated that the majority of doctor shopping is concentrated around 13% of clinicians who prescribed any opioids, and only about 2% of prescribers were used by heavy doctor shoppers [16]. Second, qualitative research indicates that information about prescriber behavior is disseminated through social networks [19].

This line of research raises an important question: Do doctor shoppers occupy distinctive structural positions in a network of patients and prescribers? In particular, we hypothesize that doctor shoppers—or high-risk individuals more generally—occupy central positions in the co-prescription network, where patients are connected to other patients if they share the same prescribers. First, by definition, they receive prescriptions from multiple prescribers and thus are likely to be connected to many other patients through those prescribers. Second, their prescribers tend to be at-risk prescribers who are targeted by other doctor shoppers. In other words, the prescribers who are connected to a doctor shopper are probably more likely to be connected to many other doctor shoppers. If doctor shoppers in isolation tend to occupy more central positions in the network, then doctor shoppers are more likely to be connected to other central nodes (other doctor shoppers), further strengthening the prominence of their structural positions. Third, simply being at a central position in the network may reflect high-risk conditions, such as being embedded in a social network of doctor shoppers or living in a community at risk for opioid misuse.

We test the hypothesis that prominence in a patient co-prescription network is an indicator of opioid misuse and related adverse outcomes using a large claims database of over 500,000 patients. Consistent with our expectations, we find that network prominence is associated with number of opioid prescriptions and risk for high morphine dosage, overdose, and opioid use disorder.

## Methodology

We use deidentified health claims from a large commercially insured population of about 19 million patients for the period of June 2015 through December 2016. Data are observed quarterly at the patient level and linked across administrative and health data. Patients are demographically representative of the US population with respect to gender and age, and representative of the commercially-insured population on all other measurable characteristics. However, because data are observational and retrospective, statistical inferences and any related conclusions should be made with caution.

We focus our study on the area most affected by the opioid crisis [20], the Appalachian region of the US. While prescription opioid misuse is a nation-wide concern, the Appalachian region of the United States has historically been the epicenter of the crisis [20, 21]. High rates of opioid prescribing early in the epidemic, economic stressors, and densely-knit social networks that facilitate drug diversion and distribution contributed to prescription drug misuse and, later, heroin initiation and abuse in Appalachia and other rural areas [22–24].

There is considerable disagreement about which states constitute Appalachia, with geographic, cultural, and political definitions providing unique but overlapping boundaries. Following Williams [25], we use the core region comprised of six states that have been included in the most influential government and scientific definitions of Appalachia–Georgia, North Carolina, Tennessee, Kentucky, Virginia, and West Virginia. Given its prominent status in the opioid epidemic (including being the probable epicenter), we add a seventh state, Ohio, which is included in the larger regional boundaries defined by the Appalachian Regional Commission [26]. To reduce the data to a manageable number of patients for SNA, we restrict our analysis sample to patients in this seven-state region who received one or more opioid prescriptions during the study period. Models using the larger ARC definition of Appalachia yield substantively identical results, but require more computing resources to converge. This process results in a sample of 526,914 patients who contribute 2,107,656 quarterly observations.

To conduct SNA, we construct a patient co-prescription network in which a tie between patients indicates that they were prescribed one or more opioids by the same prescriber (as identified with a unique provider identification number). For this process, we omit opioid agents used exclusively or primarily for medication assisted treatment (MAT; e.g., buprenorphine). This strategy reduces concerns that network centrality measures were an artifice of network clustering due to sparsely located MAT-licensed providers in medically underserved areas. Network ties are undirected and weighted by the number of unique providers from which opioid co-prescriptions (minus MATs) were obtained. For example, if Patient A and Patient B were prescribed opioids by a set of the same three unique providers, the weight of their tie is three. Ties are also pooled across three quarters (T-2, T-1, and T) to account for potential lags in information diffusion and to offset the unnatural cut points imposed by quarterly observation.

We conduct two sets of sensitivity analyses to assess the robustness of results to different network specifications. First, because the network is sparse and contains many isolates, we replicate all models after omitting patients who had no more than one unique opioid prescriber per quarter. This provides an assessment of the extent to which network prominence captures variation among moderate and high-risk patients rather than simply identifying those that are clearly not doctor shoppers. Second, we conduct sensitivity analyses using bipartite, or two-mode, network measures. This is accomplished using the generalized Co-HITS algorithm developed by Deng and colleagues [27]. This algorithm produces a PageRank score for patients that is based on both their own structural position and the prominence of the prescribers to which they are directly and indirectly connected. Many different weighting strategies are possible in the context of Co-HITS, but here we constrain all weights to be 1. In future research, we will explore different weighting strategies in attempt to improve bipartite measures of prominence in prescription networks.

### Measures

The PageRank algorithm is used to measure each patient’s prominence or influence in the co-prescription network. PageRank, originally developed to measure the importance of web pages [28], roughly measures the probability that an actor who randomly traverses the network through ties will arrive at a particular node. More specifically, PageRank is a stationary probability distribution over all nodes in a network that satisfy the following equation:

$$PR(i)=\frac{1-d}{N}+d\sum_{j\in M(i)}\frac{w_{ij}PR(j)}{s_{j}^{out}}$$

Where *PR*(*i*) is the PageRank of node *i*, *N* is the number of nodes, *d* is a damping factor (d = 0.85), *M*(*i*) is the set of the nodes that have an edge pointing to *i*, *w_ij_* is the weight of the edge from *j* to *i*, and $s_{j}^{out}$ is the strength of node *j* (the sum of the weights from node *j*). In our case, the patient-patient network does not have directed edges, and thus each undirected edge is treated as two directional edges. Although PageRank is similar to Eigenvector centrality in that both measure prominence, PageRank does not exhibit the critical localization problem of Eigenvector centrality [29].

A patient with high PageRank is someone who received opioids from health professionals who also prescribed opioids to other prominent patients (i.e., those with high PageRank). Note that a patient could have low PageRank and still visit a large number of prescribers, as long as those prescribers did not simultaneously provide opioids to many other prominent patients; Conversely, a patient could have high PageRank and still visit a small number of prescribers since PageRank takes into account the network positions of other nodes. For the current analysis, we convert the raw score for patient PageRank to a percentile value to address pronounced positive skew and to increase the interpretability of PageRank. We conduct sensitivity analyses using other specifications of PageRank and other network centralities and find that they produce similar results. These are presented in Tables 3–6.

We model four dependent variables. First, number of opioid prescriptions is a count of the number of unique prescriptions for opioids obtained in a given quarter across all prescribers. Second, overdose potential is measured using maximum daily morphine milligram equivalents (MME). MME is a value assigned to opioid medications to standardize relative potency. It was developed to assess dosing safety by facilitating calculation of the total potency of consumed drugs [30]. Daily MME is calculated by: 1) determining the total daily amount of each opioid prescribed; 2) multiplying the dose for each opioid by the CDC conversion factor; and 3) totaling MMEs for all prescriptions. We then use the maximum daily MME during a quarter to operationalize a patient’s highest risk for overdose. High MME could also be an indicator of diversion potential. In our data, 1.46% of patients in the top tenth percentile for PageRank had a maximum daily MME greater than 500 mg–over 500% of the CDC’s threshold for high overdose potential. Because doctor shopping for the purposes of diversion may not be associated with personal risk for opioid misuse outcomes (e.g., overdose, opioid use disorder), this is a potential source of unexplained variation. A binary variable is equal to 1 (else 0) if maximum daily MME is greater than 90 mg, consistent with CDC prescribing guidelines defining this as the threshold for high overdose risk [30]. Third, drug overdose is indexed using ICD-10 diagnostic codes for accidental drug poisoning in a given quarter. We calculate a measure for accidental poisoning by opioids exclusively (including synthetic opioids, e.g., fentanyl), and a separate measure that indexes poisoning by opioids or “unspecified” drugs. Although findings are consistent, we present model results based on the more inclusive measure. This decision is based on research suggesting that use of nonspecific language to classify drug poisoning leads to overuse of the “unspecified” code and undercounting of opioid overdoses [31–33]. Fourth, we create a binary indicator of opioid use disorder based on the presence of ICD-10 diagnostic codes for opioid abuse or dependence in a given quarter.

Our models also include a number of controls. We add gender (1 = female; 0 = male), age in years, and type of insurance. The latter is coded into three binary categories representing the most restrictive plans (health maintenance organizations, or HMOs), the least restrictive plans (point of service plans, or POSs), and other plans falling between these on a continuum of restrictiveness. Insurance plan is included to control for any patterns of health services utilization (i.e., which specific prescribers were accessed) that are due to plan restrictions rather than social network processes such as information sharing. To adjust for high opioid volume associated with hospice care, which may be correlated with patient PageRank percentile, we add a control for any cancer diagnosis during the study period. Finally, because our intention is to capture the relational pattern of drug seeking through prescribers rather than the sheer volume of doctor shopping, we control for each patients’ number of unique prescribers in a given quarter. This ensures that any effects of patient PageRank percentile are attributable to the position of the node in a network of co-prescription ties over and above any effect of visiting a large number of prescribers.

### Analysis

Longitudinal analyses are conducted using multivariate mixed effects logistic and negative binomial regression models with random intercepts at the person level to adjust for correlation of observations within patients over time. Models regress opioid use outcomes on network prominence (i.e., PageRank percentile) and control variables. We employ variance decomposition to model the effects of PageRank percentile on outcomes. Specifically, we split the variance in PageRank percentile into between-person and within-person estimates, where BP is the person mean (i.e., the mean value across four quarters) and WP is the difference between the current quarter and the person mean. The BP effect conveys information about how a patient’s average network prominence is associated with their average number of opioid prescriptions, for example, comparing across patients. The WP effect reflects how being more or less prominent than usual is associated with obtaining a higher or lower number of prescriptions than usual, comparing a patient to him or herself across quarters. The latter estimate is analogous to a fixed effects model, and controls for all measured and unmeasured heterogeneity at the patient level that is time invariant [34].

We also include state fixed effects to control for all unobserved heterogeneity at the state level, reducing concerns about confounding effects of differences across states in PDMP monitoring, prescription drug policies, and health care systems. Sensitivity analyses using United States Post Office city groups (based on zip code) in lieu of state fixed effects produce identical results. All models control for gender, age, type of insurance, any cancer diagnosis, and number of unique opioid prescribers. Figures of predicted counts or probabilities are presented to convey the magnitude of the effects. In figures, the y-axis range is set to +/-1 standard deviation. All data and Stata code needed to replicate these analyses will be archived in Dryad.

## Results

Our dataset contains 526,914 patients who contributed 2,107,656 quarterly prescription entries in 2016. In the patient co-prescription network, a tie between patients indicates that they were prescribed one or more opioids (excluding medication-assisted treatment) by the same prescriber. On average, each patient is connected to 29 other patients through opioid co-prescription in the same 90-day period (see Table 1). However, the degree distribution is heavily skewed and there are a small number of hub patients with very large degree (range: 0–1,178). The mean number of opioid prescriptions per quarter is 1.26 and the mean number of opioid prescribers is 0.59. About 8% of patients have high overdose potential (i.e., a max daily MME greater than 90mg) in a given quarter, and 0.16% experience an opioid overdose.

**Table 1:** Sample descriptive statistics.

	N	%	Mean	SD
*Patients (n = 526*,*914)*				
Female	309,115	58.67		
Age (years)			55.98	17.17
Insurance type				
HMO	89,173	16.92		
POS	226,903	43.06		
Other	210,838	40.01		
State				
Georgia	165,982	31.50		
Kentucky	16,333	3.10		
North Carolina	143,668	27.27		
Ohio	99,646	18.91		
Tennessee	54,388	10.32		
Virginia	43,950	8.34		
West Virginia	2,947	0.56		
*Obs (n = 2*,*107*,*656)*				
Cancer diagnosis	290,877	13.80		
Number of prescribers				
Degree			29.25	51.79
PageRank %ile			50.01	28.34
# opioid prescribers			0.59	0.75
# opioid prescriptions			1.26	2.22
Opioid use disorder	24,475	1.16		
Max daily MME>90	169,976	8.06		
Any overdose	3,360	0.16		

Table 2 provides results from the regression of number of opioids prescribed per quarter on PageRank percentile. Patients with higher PageRank are predicted to have higher numbers of opioid prescriptions compared to those with lower PageRank, controlling for the number of prescribers and other factors (See Model 1). A 10-percentile increase in between-person (BP) PageRank is associated with a predicted 18% increase in the odds of obtaining an additional opioid prescription (p < .001), adjusting for controls. Similarly, when patients have higher PageRank than usual, they also obtain more prescriptions than is typical for them. A 10-percentile increase in within-person (WP) PageRank over time is associated with a 15% increase in the odds of having an additional opioid prescription (p < .001), even after controlling for the number of unique opioid prescribers in a quarter.

**Table 2:** Mixed effects regression^1^ of opioid outcomes on between-person and within-person patient PageRank percentile and controls (n = 526,914; n obs = 2,107,656) .

	1: Num Rx		2: MME>90		3: Overdose		4: OUD	
	IRR	(CI)	OR	(CI)	OR	(CI)	OR	(CI)
Female	1.01***	(1.01–1.02)	0.54***	(0.53–0.56)	0.99	(0.92–1.08)	0.67***	(0.62–0.71)
Age (10 years)	1.06***	(1.05–1.06)	0.93***	(0.92–0.95)	1.01	(0.98–1.05)	0.72***	(0.71–0.74)
Insurance type^2^								
POS	0.80***	(0.79–0.80)	0.38***	(0.37–0.40)	0.51***	(0.45–0.58)	0.42***	(0.39–0.46)
HMO	0.92***	(0.91–0.93)	0.63***	(0.60–0.66)	0.86*	(0.77–0.97)	1.40***	(1.27–1.53)
State^3^								
Kentucky	0.94***	(0.91–0.97)	1.25	(0.97–1.63)	1.55	(0.70–3.40)	0.91	(0.57–1.45)
Virginia	0.87***	(0.85–0.90)	0.96	(0.75–1.23)	1.24	(0.58–2.66)	0.41***	(0.26–0.65)
Tennessee	0.96*	(0.93–0.99)	1.58***	(1.23–2.02)	1.35	(0.63–2.89)	3.71***	(2.40–5.74)
Ohio	0.92***	(0.89–0.95)	0.93	(0.73–1.19)	1.85	(0.87–3.92)	0.52**	(0.34–0.80)
North Carolina	1.01	(0.98–1.04)	2.44***	(1.91–3.11)	1.38	(0.65–2.93)	0.62*	(0.40–0.95)
Georgia	0.92***	(0.89–0.95)	0.81	(0.63–1.03)	1.30	(0.61–2.76)	1.03	(0.67–1.59)
Cancer diagnosis	1.02***	(1.02–1.02)	1.57***	(1.52–1.62)	2.46***	(2.26–2.67)	1.05	(0.98–1.12)
# opioid prescribers	2.27***	(2.27–2.28)	7.53***	(7.40–7.66)	1.99***	(1.92–2.07)	1.86***	(1.82–1.91)
Network prominence								
BP PageRank (10%ile)	1.18***	(1.18–1.18)	1.88***	(1.86–1.90)	1.20***	(1.18–1.23)	2.00***	(1.97–2.04)
WP PageRank (10%ile)	1.15***	(1.15–1.15)	1.27***	(1.27–1.28)	1.10***	(1.08–1.13)	1.11***	(1.09–1.12)
*ICC*	0.65		0.83		0.57		0.82	
*BIC*	4,636,263		659,547		44,533		182,155	

^1^Random intercept models adjusted for state fixed effects; incidence rate ratios or odds ratios and confidence intervals are presented

^2^Omitted category = Other

^3^Omitted category = West Virginia

* p<0.05

** p<0.01

*** p<0.001

Results from the regression of high overdose potential (>90mg maximum daily MME) on PageRank percentile are provided in Model 2 of Table 2. Patients with higher average PageRank are at greater risk for being prescribed dangerous doses of opioids compared to those with lower PageRank. A 10-percentile increase in BP PageRank predicts a 88% higher odds of having a maximum daily MME>90mg (p < .001). Also, as a patient’s own PageRank increases, so does their predicted odds of high MME, net of controls. A 10-percentile increase in PageRank predicts a 27% increased odds of overdose over time (p < .001).

Model 3 of Table 2 presents results from the regression of opioid overdose on PageRank percentile. Higher average PageRank is associated with elevated risk of overdose compared to patients with lower PageRank. A 10-percentile increase in BP PageRank predicts a 20% higher odds of overdose (p < .001). Also, as a patient’s own PageRank increases, so does their predicted odds of overdose, adjusting for number of prescribers and other control variables. A 10-percentile increase in WP PageRank over time is estimated to increase overdose risk by 10% (p < .001).

Finally, as shown in Model 4 (See Table 2), PageRank is associated with being diagnosed with an opioid use disorder (OUD). A 10-percentile increase in average PageRank predicts a 200% higher odds of OUD, comparing across patients (p < .001). At the same time, as a patient’s own PageRank increases, so too does their risk for being diagnosed with OUD. A 10-percentile increase in WP PageRank over time is associated with an 11% increase in the predicted odds of OUD.

**Fig 1:**
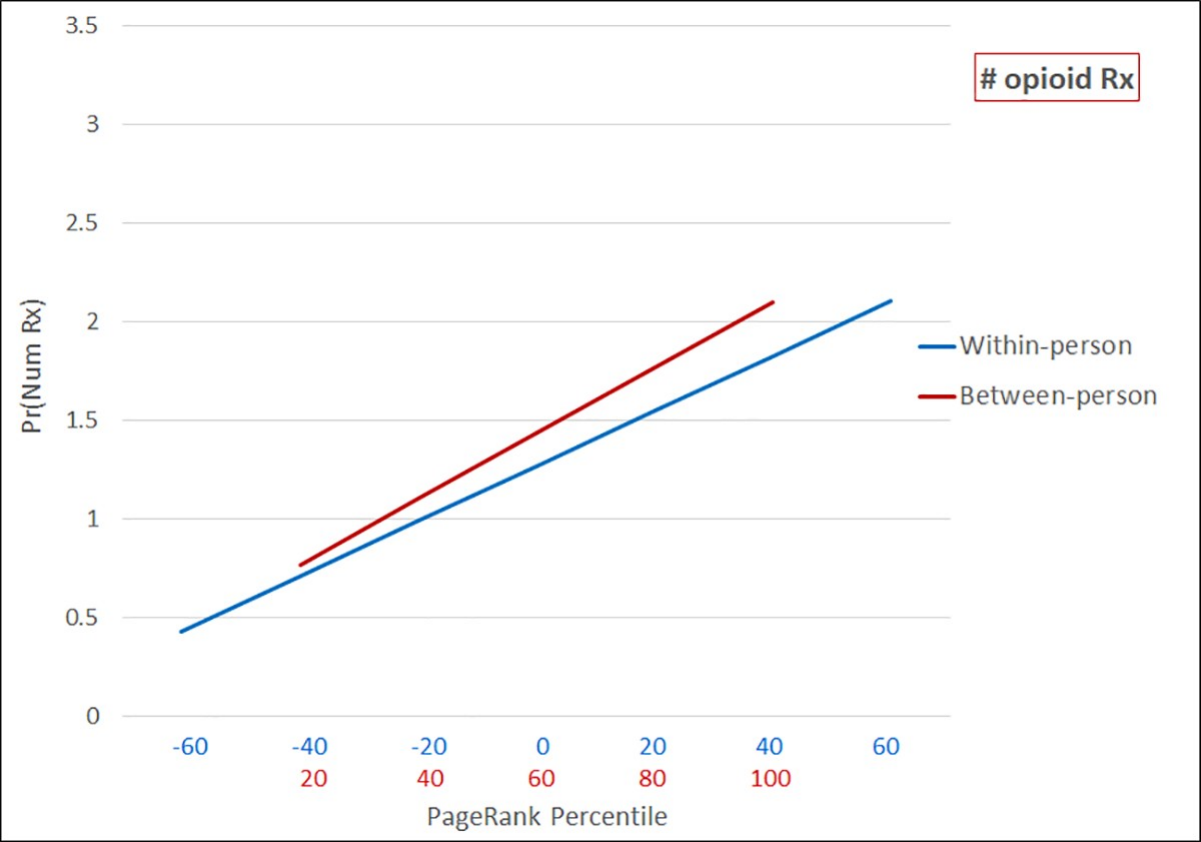
Predicted number of prescriptions as a function of within-person and between-person PageRank percentile (n = 526,914; n obs = 2,107,656).

Predicted counts or probabilities of adverse opioid outcomes as a function of network prominence are presented in figures. As shown in Fig 1, patients with the lowest PageRank percentile are predicted to obtain an average of 0.769 (CI: 0.756–0.783) opioid prescriptions per quarter, compared to 2.098 (CI: 2.082–2.113) opioid prescriptions among those with the highest PageRank percentile. Likewise, in quarters where a patient experiences an extreme decrease in PageRank percentile over time, they are predicted to obtain 0.431 (CI: 0.416–0.447) opioid prescriptions. In comparison, following a large increase in PageRank percentile, predicted number of prescriptions increases to 2.104 (CI: 2.090–2.118).

**Fig 2:**
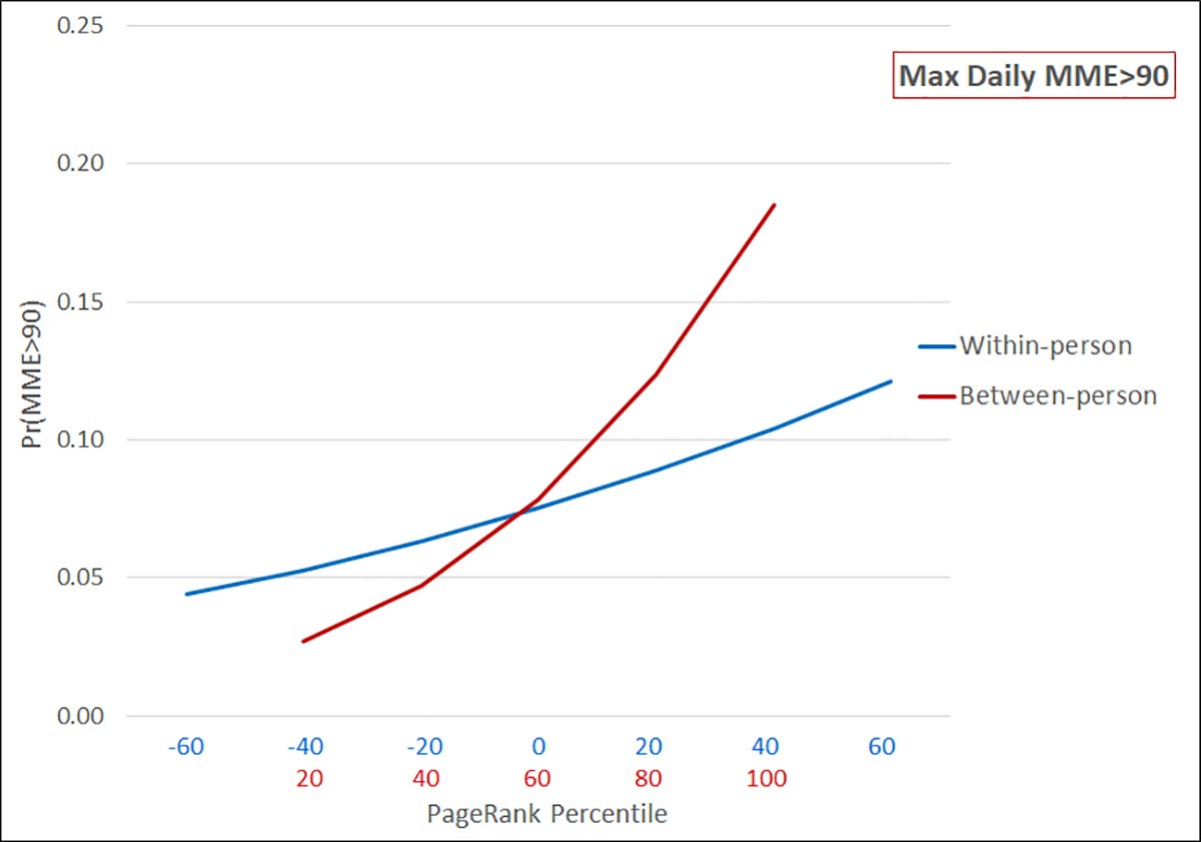
Predicted probability of MME>90 as a function of within-person and between-person PageRank percentile (n = 526,914; n obs = 2,107,656).

Predicted probabilities of maximum daily MME>90mg are presented in Fig 2. Comparing across patients, those with the lowest PageRank percentile have a predicted probability of only 0.027 (CI: 0.027–0.028) of dangerously high MME, compared to 0.185 (CI: 0.183–0.187) among those in the most prominent network positions. There is a more pronounced effect of network prominence between patients compared to within patients–a pattern consistent with the fairly high correlation of observations within patients over time (ICC = 0.83). That is, patients taking high doses of opioids tend to continue taking them over time. Nonetheless, the predicted probability of high MME ranges from 0.044 (CI: 0.043–0.045) when a person experiences a large decrease in PageRank percentile to 0.121 (CI: 0.120–0.123) in quarters when they experience the greatest increase over time.

**Fig 3:**
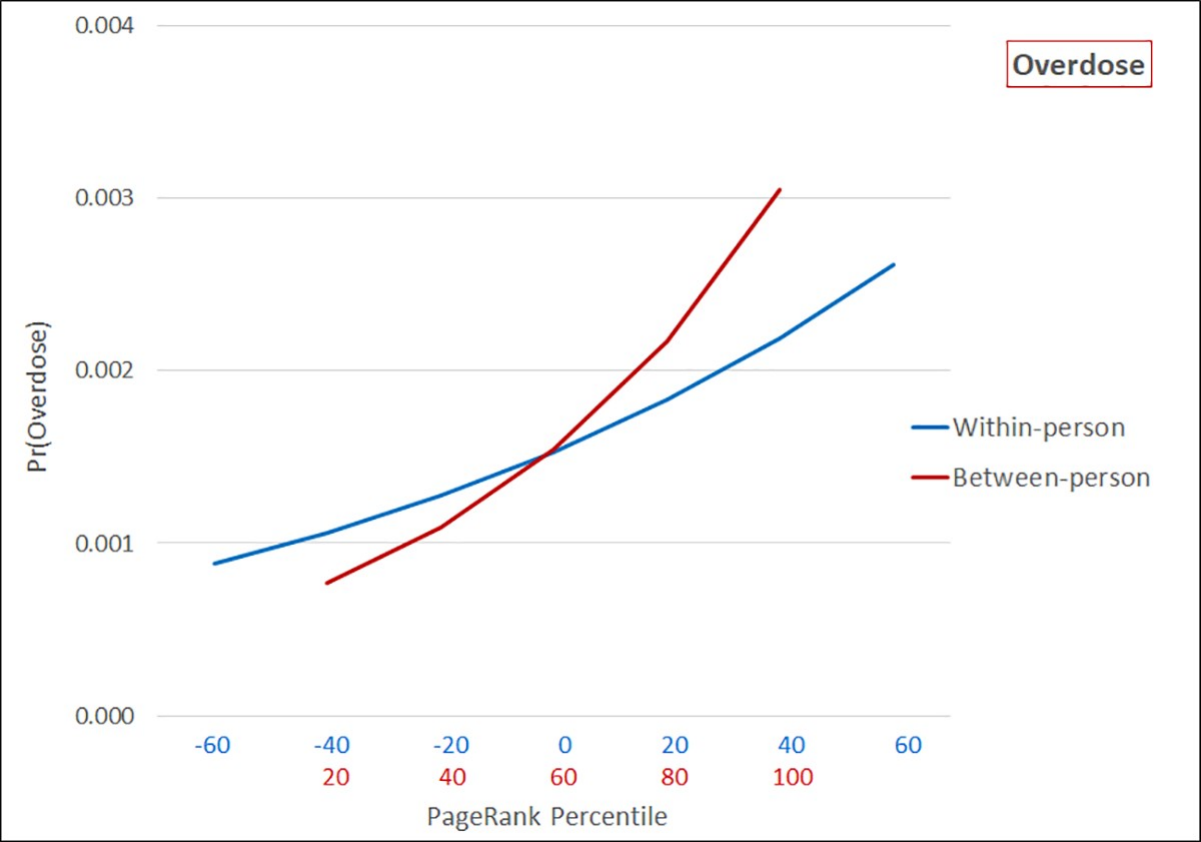
Predicted probability of overdose as a function of within-person and between-person PageRank percentile (n = 526,914; n obs = 2,107,656).

Fig 3 presents predicted probabilities of opioid overdose. A patient with the lowest PageRank percentile has a predicted probability of overdose of 0.00077 (CI: 0.00070–0.00084) relative to 0.00305 (CI: 0.00281–0.00328) for those with the highest average PageRank. Over time, a patient experiencing a large decrease in PageRank percentile compared to usual is expected to have a 0.00089 (CI: 0.00075–0.00102) probability of overdose, while predicted probability of overdose is 0.00262 (CI: 0.00229–0.00294) when increases in PageRank are large.

**Fig 4:**
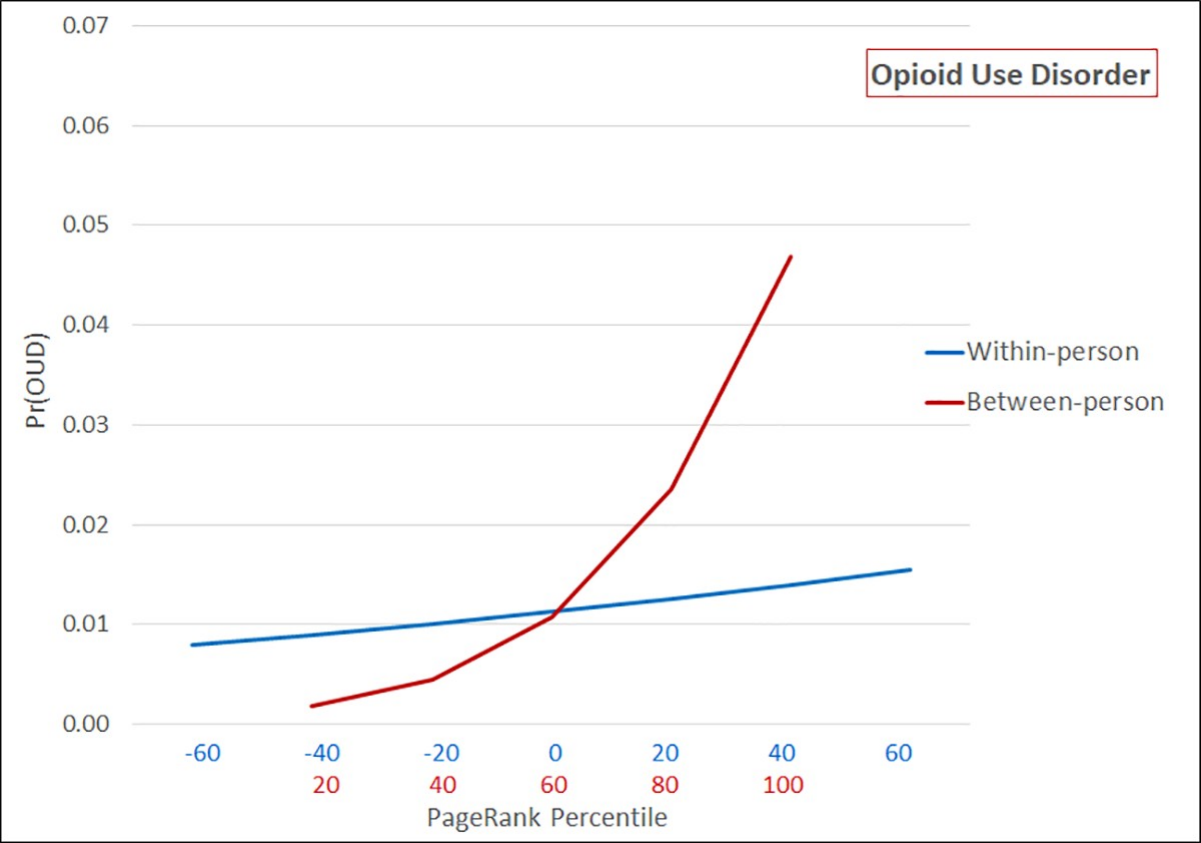
Predicted probability of opioid use disorder as a function of within-person and between-person PageRank percentile (n = 526,914; n obs = 2,107,656).

Finally, comparing across patients, those with the lowest PageRank percentile have a predicted probability of OUD of 0.0018 (CI: 0.0017–0.0019), compared to 0.0468 (CI: 0.0451–0.0485) among those in the most prominent network positions (See Fig 4). Like high dosage prescription regimens, OUD is highly correlated over time within patients (ICC = 0.82). Consequently, there is a more pronounced effect of network prominence between patients compared to within patients. The predicted probability of receiving a diagnosis of OUD ranges from 0.0080 (CI: 0.0076–0.0084) when a person experiences a large decrease in PageRank percentile to 0.0155 (CI: 0.0149–0.0162) in quarters when they experience the greatest increase over time.

Results from sensitivity analyses are presented in Tables 3–6. First, we replicate all models after omitting patients who had no more than one unique opioid prescriber per quarter. Findings are consistent with those using the full sample (See Table 3), suggesting that network prominence may be useful for distinguishing between gradations of moderate to high-risk patient behavior. Specifically, within-person and between-person PageRank percentile are significantly associated with number of opioid prescriptions, high-risk MME volume, overdose, and opioid use disorder. Findings are smaller in magnitude in the restricted samples, as would be expected with reduced variation to explain, but the direction and significance of effects are robust. Second, we replicate models using a variety of different methods for operationalizing network centrality. These include employing a bipartite network of patients and prescribing physicians in lieu of a projected one-mode network of patients (See Table 4); standardized raw PageRank rather than PageRank percentile (See Table 5); and logged degree centrality instead of PageRank (See Table 6). All of these findings broadly provide support for a network approach to measuring drug seeking. That is, network metrics are significantly and positively associated with adverse drug use outcomes across all models.

**Table 3:** Mixed effects regression^1^ of opioid outcomes on between-person and within-person patient PageRank percentile and controls among high-risk patients with more than one prescriber per quarter (n = 68,401; n obs = 273,604).

	1: Num Rx		2: MME>90		3: Overdose		4: OUD	
	IRR	(CI)	OR	(CI)	OR	(CI)	OR	(CI)
Female	1.00	(0.99–1.01)	0.42***	(0.39–0.46)	1.00	(0.88–1.14)	0.78***	(0.71–0.85)
Age(10 years)	0.99***	(0.99–1.00)	0.62***	(0.60–0.64)	0.92**	(0.87–0.97)	0.69***	(0.66–0.71)
Insurance type^2^								
POS	0.85***	(0.84–0.87)	0.31***	(0.28–0.35)	0.66***	(0.54–0.79)	0.74***	(0.65–0.83)
HMO	0.88***	(0.87–0.89)	0.47***	(0.43–0.52)	0.81*	(0.69–0.96)	1.12*	(1.00–1.25)
State^3^								
Kentucky	0.93	(0.86–1.01)	1.51	(0.79–2.88)	0.87	(0.54–1.41)	1.16	(0.53–2.56)
Virginia	0.91**	(0.84–0.98)	3.51	(2.76–4.47)	1.04	(0.80–1.36)	0.48	(0.22–1.04)
Tennessee	0.92*	(0.85–0.99)	1.40	(1.10–1.78)	0.97	(0.77–1.22)	3.92***	(1.85–8.29)
Ohio	0.98	(0.91–1.05)	2.14	(1.67–2.73)	1.33**	(1.09–1.60)	0.54	(0.26–1.15)
North Carolina	1.11**	(1.03–1.19)	1.23	(0.96–1.58)	1.08	(0.93–1.26)	0.75	(0.36–1.59)
Georgia	0.91*	(0.85–0.98)	0.74	(0.39–1.41)	[omitted]	1.26	(0.60–2.65)
Cancer diagnosis	1.03***	(1.02–1.03)	1.51***	(1.43–1.59)	2.50***	(2.20–2.84)	1.05	(0.97–1.14)
# opioid prescribers	1.35***	(1.35–1.35)	2.89***	(2.83–2.96)	1.49***	(1.41–1.56)	1.29***	(1.26–1.33)
Network prominence								
BP PageRank (10%ile)	1.04***	(1.04–1.04)	1.68***	(1.64–1.73)	1.08**	(1.03–1.13)	1.45***	(1.40–1.50)
WP PageRank (10%ile)	1.04***	(1.04–1.05)	1.20***	(1.18–1.21)	1.09**	(1.03–1.14)	1.14***	(1.11–1.16)
*ICC*	0.71		0.85		0.54		0.73	
*BIC*	1,115,970		218,590		17,629		79,097	

^1^Random intercept models adjusted for state fixed effects; incidence rate ratios or odds ratios and confidence intervals are presented

^2^Omitted category = Other

^3^Omitted category = West Virginia

* p<0.05

** p<0.01

*** p<0.001

**Table 4:** Mixed effects regression^1^ of opioid outcomes on between-person and within-person patient bipartite PageRank percentile and controls (n = 526,914; n obs = 2,107,656).

	1: Num Rx		2: MME>90		3: Overdose		4: OUD	
	IRR	(CI)	OR	(CI)	OR	(CI)		(CI)
Female	1.02***	(1.01–1.02)	0.58***	(0.57–0.60)	1.00	(0.92–1.08)	0.70***	(0.66–0.75)
Age(10 years)	1.06***	(1.06–1.06)	0.96***	(0.95–0.97)	1.02	(0.98–1.05)	0.77***	(0.75–0.79)
Insurance type^2^								
POS	0.75***	(0.74–0.75)	0.30***	(0.29–0.32)	0.47***	(0.42–0.53)	0.32***	(0.29–0.35)
HMO	0.92***	(0.91–0.92)	0.63***	(0.60–0.66)	0.85**	(0.76–0.96)	1.29***	(1.18–1.41)
State^3^								
Kentucky	1.00	(0.97–1.04)	1.51**	(0.80–3.84)	1.75	(0.80–3.84)	1.13	(0.72–1.77)
Virginia	0.95**	(0.92–0.98)	1.23	(0.96–1.58)	1.47	(0.69–3.15)	0.60*	(0.39–0.93)
Tennessee	1.07***	(1.04–1.11)	2.14***	(1.67–2.73)	1.70	(0.80–3.64)	5.63***	(3.69–8.59)
Ohio	1.05**	(1.02–1.09)	1.40**	(1.10–1.78)	2.33*	(1.10–4.95)	0.93	(0.61–1.41)
North Carolina	1.16***	(1.13–1.20)	3.51***	(2.76–4.47)	1.88	(0.89–3.99)	1.18	(0.78–1.80)
Georgia	1.13***	(1.10–1.17)	1.50***	(1.18–1.92)	1.95	(0.92–4.14)	2.55***	(1.67–3.87)
Cancer diagnosis	0.99***	(0.98–0.99)	1.41***	(1.36–1.44)	2.28***	(2.09–2.48)	0.95	(0.89–1.01)
# opioid prescribers^4^	2.41***	(2.41–2.42)	11.07***	(10.86–11.27)	1.97***	(1.90–2.05)	2.27***	(2.21–2.33)
Network prominence								
BP Bip PageRank (10%ile)	1.10***	(1.10–1.10)	1.27***	(1.25–1.28)	1.21***	(1.18–1.23)	1.47***	(1.45–1.50)
WP Bip PageRank (10%ile)	1.12***	(1.12–1.12)	1.08***	(1.07–1.08)	1.11***	(1.08–1.14)	1.02**	(1.01–1.03)
*ICC*	0.65		0.83		0.57		0.83	
*BIC*	4,703,998		677,120		44,565		187,576	

^1^Random intercept models adjusted for state fixed effects; incidence rate ratios or odds ratios and confidence intervals are presented

^2^Omitted category = Other

^3^Omitted category = West Virginia

^4^Number of prescribers truncated at 4 in Model 2 to allow convergence

* p<0.05

** p<0.01

*** p<0.001

**Table 5:** Mixed effects regression^1^ of opioid outcomes on between-person and within-person patient standardized PageRank and controls (n = 526,914; n obs = 2,107,656).

	1: Num Rx		2: MME>90		3: Overdose		4: OUD	
	IRR	(CI)	OR	(CI)	OR	(CI)	OR	(CI)
Female	1.02***	(1.01–1.02)	0.55***	(0.54–0.57)	1.00	(0.92–1.09)	0.68***	(0.64–0.72)
Age(10 years)	1.06***	(1.06–1.06)	0.95***	(0.94–0.96)	1.03	(0.99–1.06)	0.77***	(0.75–0.78)
Insurance type^2^								
POS	0.79***	(0.78–0.79)	0.37***	(0.35–0.39)	0.49***	(0.44–0.56)	0.40***	(0.36–0.44)
HMO	0.92***	(0.91–0.92)	0.62***	(0.59–0.65)	0.85**	(0.76–0.96)	1.30***	(1.19–1.43)
State^3^								
Kentucky	0.93***	(0.90–0.97)	1.23	(0.96–1.59)	1.56	(0.71–3.43)	0.90	(0.57–1.42)
Virginia	0.87***	(0.84–0.89)	0.91	(0.71–1.16)	1.24	(0.58–2.67)	0.40***	(0.26–0.62)
Tennessee	0.94***	(0.91–0.97)	1.45**	(1.13–1.84)	1.34	(0.63–2.89)	3.32***	(2.17–5.08)
Ohio	0.92***	(0.89–0.95)	0.90	(0.71–1.14)	1.87	(0.88–3.99)	0.52**	(0.34–0.80)
North Carolina	0.97	(0.94–1.01)	2.11***	(1.66–2.68)	1.36	(0.64–2.90)	0.55**	(0.36–0.84)
Georgia	0.89***	(0.87–0.92)	0.73**	(0.57–0.92)	1.28	(0.60–2.73)	0.92	(0.60–1.40)
Cancer diagnosis	1.02***	(1.02–1.03)	1.53***	(1.49–1.58)	2.47***	(2.27–2.69)	1.05	(0.98–1.12)
# opioid prescribers^4^	2.49***	(2.49–2.50)	8.81***	(8.65–8.97)	2.07***	(1.98–2.15)	1.83***	(1.78–1.87)
Network prominence								
BP Std PageRank	1.32***	(1.32–1.33)	9.61***	(9.21–10.03)	1.31***	(1.25–1.36)	4.02***	(1.06–1.77)
WP Std PageRank	1.13***	(1.13–1.13)	1.21***	(1.16–1.26)	1.07*	(1.01–1.13)	1.18***	(1.13–1.32)
*ICC*	0.60		0.83		0.59		0.83	
*BIC*	4,724,401		663,720		44,725		183,534	

^1^Random intercept models adjusted for state fixed effects; incidence rate ratios or odds ratios and confidence intervals are presented

^2^Omitted category = Other

^3^Omitted category = West Virginia

^4^Number of prescribers truncated at 4 in Model 2 to allow convergence

* p<0.05

** p<0.01

*** p<0.001

**Table 6:** Mixed effects regression^1^ of opioid outcomes on between-person and within-person patient logged degree centrality and controls (n = 526,929; n obs = 2,107,716).

	1: Num Rx		2: MME>90		3: Overdose		4: OUD	
	IRR	(CI)	OR	(CI)	OR	(CI)	OR	(CI)
Female	1.02***	(1.02–1.03)	0.56***	(0.55–0.58)	1.00	(0.92–1.09)	0.69***	(0.65–0.74)
Age(10 years)	1.05***	(1.05–1.05)	0.91***	(0.90–0.92)	1.01	(0.97–1.04)	0.70***	(0.68–0.72)
Insurance type^2^								
POS	0.85***	(0.84–0.85)	0.47***	(0.45–0.50)	0.54***	(0.48–0.61)	0.53***	(0.49–0.59)
HMO	0.92***	(0.91–0.93)	0.64***	(0.6–0.67)	0.86*	(0.77–0.97)	1.47***	(1.33–1.61)
State^3^									
Kentucky	0.82***	(0.80–0.85)	0.77	(0.60–1.01)	1.39	(0.63–3.04)	0.62*	(0.39–0.98)
Virginia	0.72***	(0.70–0.74)	0.48***	(0.38–0.62)	1.05	(0.49–2.25)	0.23***	(0.15–0.36)
Tennessee	0.73***	(0.71–0.76)	0.61***	(0.47–0.78)	1.06	(0.50–2.27)	1.57*	(1.02–2.43)
Ohio	0.71***	(0.68–0.73)	0.37***	(0.29–0.47)	1.49	(0.70–3.16)	0.23***	(0.15–0.35)
North Carolina	0.67***	(0.65–0.69)	0.58***	(0.45–0.74)	0.96	(0.45–2.04)	0.16***	(0.10–0.24)
Georgia	0.54***	(0.52–0.55)	0.12***	(0.09–0.15)	0.81	(0.38–1.72)	0.17***	(0.11–0.27)
Cancer diagnosis	1.03***	(1.03–1.04)	1.64***	(1.59–1.69)	2.51***	(2.31–2.74)	1.10**	(1.03–1.17)
# opioid prescribers	2.26***	(2.26–2.27)	7.62***	(7.49–7.75)	2.04***	(1.97–2.11)	1.88***	(1.83–1.92)
Network prominence								
BP degree logged	1.41***	(1.41–1.42)	3.58***	(3.52–3.64)	1.40***	(1.35–1.45)	3.68***	(3.57–3.79)
WP degree logged	1.40***	(1.39–1.40)	1.93***	(1.90–1.96)	1.22***	(1.17–1.28)	1.33***	(1.30–1.37)
*ICC*	0.65		0.83		0.57		0.82	
*BIC*	4,581,141		652,846		44,502		181,025	

^1^Random intercept models adjusted for state fixed effects; incidence rate ratios or odds ratios and confidence intervals are presented

^2^Omitted category = Other

^3^Omitted category = West Virginia

* p<0.05

** p<0.01

*** p<0.001

## Conclusions

In this study, we examine whether structural position in a co-prescription network could provide insight into high-risk drug-seeking. We find that patients in positions of prominence in a co-prescription network disproportionately experience adverse opioid outcomes, even after controlling for the number of unique opioid prescribers visited. Moreover, as a patient’s own prominence increases over time, so does their risk for these outcomes, compared to their own average level of risk. These results are consistent with a pattern of information sharing among networked, drug-seeking patients about effective targets for doctor shopping [19], or localized prescription drug diversion coalitions [35]. Alternatively, observable attributes (e.g., being located in a pain clinic, being isolated from other providers) may make particular prescribers vulnerable to doctor shopping, even in the absence of direct information sharing.

Our findings have important implications for evolving social responses to policy change. Specifically, relationships between network prominence and risk for opioid misuse and overdose were not attributable to the sheer number of prescribers, as this variable was held constant in regression models. Rather, in characterizing doctor shopping behavior, our findings indicate that *which prescribers* a patient targets (i.e., their relative network centrality) may be as critical as *how many*. Supply-side interventions to reduce prescription drug misuse (e.g., prescription limits and guidelines, mandatory prescription monitoring, prescriber incentives to reduce volume) have typically used the latter approach to measure and mitigate fraud and abuse [36, 37]. Thus, cooperation and information sharing may increasingly be essential strategies for procuring opioids in today’s policy environment. While existing research has focused on the turn toward black-market alternatives to prescription opioids (e.g., heroin, fentanyl) [22, 38–40], our findings highlight collaborative and calculated doctor shopping as another potential behavioral response to supply-side interventions. If true, social network analysis is likely to become an increasingly essential tool for characterizing prescription drug misuse, including diversion.

A limitation of our analysis is that social mechanisms underlying network structure are not directly observed and must be inferred. However, it is reassuring that findings are robust to different specifications of network centrality, and that we are able to rule out alternative explanations for network clustering, including cancer-related pain management, shortages of licensed medication-assisted therapy (MAT) prescribers, type of insurance, and state or county of residence. Also, because our data are derived from claims billed through commercial insurance carriers, we are unable to observe cash transactions and Medicaid claims. Since self-payment is a strategy for avoiding detection of doctor shopping behavior [41], our findings may underestimate the effects of social network prominence and should be replicated using PDMP data.

In sum, while existing research suggests that social mechanisms facilitate doctor shopping, we are aware of no prior large-scale analysis using social network methods to characterize this behavior. These results are significant because they underscore the potential of network approaches to improve measurement, and also to expose social dynamics underlying the opioid epidemic that are not discoverable with traditional threshold approaches (e.g., number of prescribers). Future research should explore these possibilities. For example, the predictive value of social network indicators of doctor shopping should be tested against traditional measures for identifying early and intermittent opioid misuse, or for distinguishing high-volume personal use from fraud and diversion. A network approach might also be used to identify social or geographic “hot spots” for intervention (e.g., increased harm reduction efforts) or for early prediction of unmet substance abuse treatment need.
